# Nursing students’ attitudes toward intimate partner violence and its relationship with self-esteem and self-efficacy

**DOI:** 10.1186/s12912-024-01868-2

**Published:** 2024-03-27

**Authors:** Rania Rabie El-Etreby, Warda Elshahat Hamed, Eman Sameh AbdElhay, Nesma Ahmed Kamel

**Affiliations:** Psychiatric Nursing and Mental Health, Faculty of Nursing, Mansoura University, Mansoura, Egypt

**Keywords:** Nursing students, Intimate partner violence, Self-esteem, Self-efficacy

## Abstract

Understanding nursing students’ attitudes toward Intimate Partner Violence (IPV) is pivotal because it may impact the care and support, they provide victims. This study aimed to explore nursing students’ attitudes toward intimate partner violence and its relationship with self-esteem and self-efficacy. A cross-sectional descriptive research design was used in the current study in the faculty of nursing at Mansoura University, Egypt. A total of 1322 nursing students participated in the study. Data were collected from April to June 2023 by using various tools: sociodemographic interviews and academic data profiles, the Intimate Partner Violence Attitude Scale (IPVAS)—Revised, *the Rosenberg Self-Esteem Scale (RSE) (*Arabic version), and the General Self-Efficacy Scale. Results: The findings show the distribution of the Intimate Partner Violence Attitude Scale (IPVAS), with 78.7% of nursing students disapproving of the abuse domain, 72.6% (approving of the violence domain) and 34.2% approving of the control domain. 65.8% had a moderate level of self-esteem, and 62.8% had a low level of self-efficacy. The study concluded that self-esteem and self-efficacy are significant in shaping attitudes toward intimate partner violence; higher levels of self-esteem and self-efficacy are associated with lower approval for intimate partner violence. Further research is needed to explore the factors that contribute to attitudes and levels of self-esteem and self-efficacy among nursing students. It would also be useful to study how education and training affect these attitudes.

## Introduction

Intimate partner violence (IPV) is a global public health issue that adversely affects the mental and physical health of people of all backgrounds, including university students [1]. Studies suggest that intimate partner violence IPV affects 30% of women worldwide and accounts for 35% of women’s homicides. Women suffer the majority burden of IPV globally [1, 2]. In Arab nations, 11% and 45% of university students have reported experiencing physical violence, with 31% of Egyptian university students reporting different types of IPV [3].Based on a survey conducted by theBarometer Arab [4], 90% of women in Egypt aged 17–28 and 85% of those aged 29–40 stated that they had encountered instances of sexual harassment. Furthermore, nearly half (48%) of Egyptian women reported experiencing domestic violence. Additionally, certain studies suggest that 9 out of 10 women between the ages of 15 and 49 have been subjected to female genital mutilation (FGM), as indicated by data from the Ministry of Health and Population in 2015 [5].

Intimate partner violence (IPV) is any physical, sexual, psychological, or controlling behavior inflicted on a person by their intimate partner. Although both genders can be victims of IPV, studies indicate that men are usually the perpetrators. At the same time, women tend to suffer more physical, sexual, or violent abuse in the hands of their partners [1]. Therefore, intimate partner violence (IPV) among young people and emerging adults is widespread and has a variety of adverse impacts on one’s psychological and physical health [6]. Physical intimate partner violence IPV and sexual violence victimization in male or female adolescents or emerging adults have been linked to sexual risk behaviors, violence-related behaviors, substance use, poor mental health, other health risk behaviors, and poor health status, according to numerous studies [7–9]. Additionally, student victim survivors may suffer poor academic effects, such as higher academic challenges, reduced academic achievement, and academic disengagement, in addition to the effects of violence on their physical and mental health [10, 11].

In the context of this study, self-efficacy is considered a distinct variable encompassing an individual’s beliefs regarding their capabilities within interpersonal relationships. It refers to an individual’s confidence in their capacity to formulate, modify, and implement effective action plans for navigating potential life events [12]. According to Raghavan [13], a self-efficacy relationship encompasses the person’s potential to handle disagreements in romantic relationships effectively, come to an understanding with the partner, and stand up for their rights. Self-efficacy is one of the notions of empowerment, and studies have shown that people need it. According to Zarei [14], one’s perception of one’s ability to carry out a successful behavior is known as self-efficacy. In contrast, people with poor self-efficacy struggle to deal with issues that arise during violent acts or cannot stop intimate partner violence, which they are expected to experience in the future. The chance of being exposed to violence rises, and the quality and duration of romantic relationships are impacted [15].

Self-esteem, a belief system of self-efficacy, motivation, and self-regulation, moderates the relationship between the adverse effects of intimate partner violence (IPV) exposure and psychological health problems in those exposed to it. One’s self-esteem is permanently damaged by repeated exposure to physical and psychological IPV [16–18].

Researches on the prevalence of intimate partner violence among nurses and nursing students, concluded that the prevalence of intimate partner violence was unacceptably high, with results of 48.2% in nursing students and 58.7% for nurses [19]. The decrease in the IPV among nursing students could be regarded to their lack of awareness of the warning signs of intimate partner violence as they may be experiencing an abusive relationship themselves [20].

According to Penado Abilleira and Rodicio-García [21] exposure to intimate partner violence (IPV) has a notable impact on the affective self-concept, ultimately leading to a compromised physical self-concept. IPV not only adversely influences self-esteem but also diminishes women’s belief in their capacity to effectively execute behaviors to attain desired outcomes, referred to as self-efficacy [12]. Previous research has indicated that IPV is correlated with a decrease in self-efficacy, and lower levels of self-esteem relate to more severe depressive symptoms in victims of IPV [22, 23].

Examining nursing students’ attitudes toward intimate partner violence (IPV) and how it relates to their self-esteem and self-efficacy is crucial in Egypt. This is because Egypt, like many other countries, is struggling to combat the prevalence and effects of IPV. In addition, studying nursing students’ attitudes in Egypt offers valuable insights into the country’s culture and society. By examining how cultural norms, social perceptions, and gender roles affect responses to intimate partner violence (IPV), we can create practical and culturally sensitive strategies and interventions to address this issue in Egypt. According to El-Safty [24], violence has become ingrained in the culture to the extent that both victims and perpetrators may not identify the behavior as abusive. The violence is normalized and routinized, shielded from scrutiny and opposition by its deep roots in tradition, religion, or societal norms. Critically, any effort to introduce alternative perspectives that challenge established beliefs and practices faces strong resistance, being labeled as foreign and culturally insensitive in the context of Egyptian society. This underscores the significance of the current research and similar studies. Hence, understanding intimate partner violence and its influences on the attitudes and behaviors of young adults, particularly university students, is crucial. It is essential for them to be aware of the significant effects of such violence and to develop their own self-esteem and self- efficacy, contributing to their overall maturity and productivity. In addition, valuable insights can be gained for developing effective interventions and educational programs by examining nursing students’ attitudes toward intimate partner violence who will become healthcare providers. many studies have examined violence toward women in Egypt [3, 25, 26], however in our research, we differed from the prevalent focus by specifically examining intimate partner violence within the context of undergraduate nursing students. Additionally, we introduced novel elements to the study, incorporating self-esteem and self-efficacy as new variables. Therefore, this study aimed to explore nursing students ‘attitudes toward intimate partner violence and its relationship with self-esteem and self-efficacy. The main questions of this study were as follows: What are nursing students’ attitudes toward intimate partner violence? How do nursing students view their self-esteem and their self-efficacy? Is there a correlation between nursing students’ self-esteem and self-efficacy and their attitudes toward intimate partner violence?

## Methods

### Study design and sample

A cross-sectional descriptive correlational design was used for this study. It took place at Mansoura University’s Faculty of Nursing, which adhered to Egyptian nursing education regulations and was under the direction of Egypt’s Ministry of Higher Education. A convenience sample of 1322 students from the bachelor program’s first, second, third, and fourth years was chosen for their appropriateness and willingness to participate and absence of physical, mental, or cognitive impairments.

### Sample size calculation

The sample size was obtained using G-power software V.3.1.9.7 and data from the literature [27] by applying the following formula: using a 5% significance criterion and an 80% research power: n = where Z1-/2 = is the standard normal variate; 1.96 for 5% type 1 error; SD is the standard deviation of the variable; and d is the absolute error or precision. Therefore, *n* = 1115.4. The research requires a sample size of 1116. Our sample size (1322) exceeded the minimum (1116). Consequently, we declare that our sample size is statistically significant and that our findings will be dependable.

### Ethical consideration

The research gained approval for conduct from the Research Ethics Committee of the Faculty of Nursing, Mansoura University, which adheres to the World Medical Association’s Code of Ethics (Declaration of Helsinki). The anonymous online poll was interpreted as consent. The anonymity of the participants was maintained throughout the investigation. Any personal information gathered was kept secret, and only the study team could access it.

#### Data Collection

The following tools were used in the study.

#### Student’s sociodemographic and academic data profile.”

Included: students’ sociodemographic characteristics, such as age, gender, and marital status, as well as their academic data, including their GPA and year of study. This tool provided valuable background information about the participants and helped us understand any potential confounding variables that may influence the results.

#### Intimate partner violence attitude scale (IPVAS)—revised

***The*** IPVAS is a 17-point assessment test to measure undergraduate students’ attitudes toward violence against intimate partners. The scale was created by Smith [28]. It measures attitudes on a range from “strongly disagree (1)” to “strongly agree (5).” The scale has three categories: Control, which assesses beliefs about social control and monitoring a partner’s behavior; Violence, which investigates attitudes toward physical abuse and threats of physical abuse; and Abuse, which examines the acceptability of verbal and nonverbal abusive behaviors. The item scores are weighted and combined to produce the three subscale scores. The scale has seven questions with reverse-scored responses. Smith [28] obtained alpha coefficient values for subscales ranging from 0.69 to 0.81, and their findings confirmed the anticipated dimensionality. The Cronbach’s alpha reliability coefficient of the scale was determined to be 0.74 Fincham [29]. When scores are high, there is a favorable attitude toward using violence in relationships. In addition, the researchers conducted an exploratory factor analysis to evaluate the scale’s validity. Before rotation, the loadings varied between 0.490 and 0.890; however, after applying the post-varimax rotation, the loadings ranged from 0.510 to 0.911, above the criterion of 0.320. The combined loadings explained 74.22% of the total variation. Furthermore, the Kaiser-Meyer-Olkin measure produced a score of 0.910, indicating that the data were appropriate for factor analysis. Bartlett’s test of sphericity yielded a statistically significant result (*P* = 0.000), indicating that the correlation matrix is suitable for factor analysis. Therefore, it is justified to keep all the items on this scale.

#### Rosenberg self-esteem scale (RSE): arabic version

The Self-esteem Scale was employed in this research to assess students’ self-esteem [30]. It is a standardized, brief, structured, self-report questionnaire with ten items. All are graded on a four-point scale, ranging from strongly agreeing to strongly disagreeing. Items 2, 5, 6, 8, and 9 are negative, whereas items 1, 3, 4, 7, and 10 are positive. To limit the influence of the respondent set, the positive and negative items were given in random order. The higher the score, the higher the self-esteem. Internal consistency varies from 77 to 88, and test-retest reliability extends from.82 to.85 [31].

#### The general self-efficacy scale

The scale was developed by Schwarzer and Jerusalem [32] to predict coping and adaptation to everyday difficulties and other types of stressful life events. It is intended to examine positive self-belief and to address a wide range of life circumstances. The GES consists of ten items that are evaluated on a four-point Likert scale, ranging from 1 (Not at all true) to 4 (Exactly true). The Arabic version of the scale used in the current study has been validated by Crandall [33]. A total score on a scale of 10 to 40 or an average scale score on a range of 1 to 4 may be computed. Higher scores indicate a higher level of perceived general self-efficacy, whereas lower values indicate a lower level of perceived general self-efficacy. Cronbach’s alpha ranges from 0.76 to 0.90 [32].

### Validity and reliability

At first, the scale on intimate partner violence (IPV-R) was translated into Arabic using a forward and backward translation method. The translated questionnaire was then adapted to fit Arabic cultural norms. Two highly proficient native Arabic speakers who are accomplished academics in the fields of psychiatry and mental health nursing, and hold the academic status of Full Professor translated the questionnaire from English to Arabic. An English-language expert who is fluent in Arabic back-translated the Arabic version. Native Arabic speakers who were not involved in the translation process verified the final translation. The forward-to-back translation process was repeated until the comparative findings matched exactly. The questionnaire was then given to three Arabic psychiatric nursing professionals, who provided their opinions on its importance, relevance, and simplicity. To evaluate the reliability of the study tools, a test-retest procedure was conducted on a sample of 10% nursing students with a two-week interval to examine their stability over time and to achieve face and content validity by defining and assessing fit in several categories [34]. Preliminary research findings were not included in the final study results. The tools’ reliability was tested using Cronbach’s alpha test, with the Intimate Partner Violence Attitude Scale (IPVAS) scoring a value of 0.90, the Rosenberg Self-Esteem Scale (RSE) scoring 0.89, and the General Self-Efficacy Scale scoring 0.89. Additionally, a confirmatory factor analysis was carried out to validate the content of the IPV scale after translation.

### Data collection procedure

Authorization to translate the Intimate Partner Violence (IPV) scale into Arabic was obtained from the scale developer. Data collection was conducted over a period of approximately three months, spanning from April to June 2023. The study employed an online data collection instrument, specifically a Google Form spreadsheet, for the survey process. Invitations to participate in the study were disseminated via email to selected students, including a hyperlink to the online questionnaire. The researchers utilized students’ e-mails to describe the components of the tools and how to complete the online questionnaire.

### Statistical analysis

The gathered data were systematically arranged and subjected to analysis through SPSS version 20.0 for Windows (SPSS, Chicago, IL). Continuous data were normally distributed and were expressed in mean ± standard deviation (SD). Categorical data were expressed in number and percentage. Correlation co-efficient test was used to test for correlations between the intimate partner violence attitude scale, self-esteem, and self-efficacy levels. The linear regression analysis model was used to determine the factors that predict intimate partner violence attitude scores. Additionally, the internal consistency (reliability) of the research questionnaires was assessed. The chosen statistical significance level was set at *p* < 0.05.

## Results

### The sociodemographic characteristics of nursing students

The total number of participant students was 1322. The mean age of the students was 20.65 (SD = 1.40). More than half, 55.8%, were female, while 44.2% were male. Regarding the academic year, 23.9% were in their first year, 12.0% were in their second year, 34.6% were in their third year, and 29.5% were in their fourth year. Furthermore, the majority of them (93.6%) were single. More than three-quarters (77%) reported no exposure to violence, while 10.1% reported being exposed to violence. Regarding witnessing violence, two-thirds (66.6%) report no witnesses, while 15.9% report witnessing violence. Regarding the types of violence experienced by the students, 65.9% did not experience violence, 12.6% experienced verbal violence, 9.9% experienced physical violence, and9.2%experienced physical violence (Table 1).

### The mean score of the intimate partner violence attitude scale (IPVAS) among nursing students

The data suggest that nursing students had varying attitudes toward intimate partner violence, with some exhibiting lower scores in the abuse domain and higher scores in the violence and control domains. The mean score for the abused domain was 7.51, with an SD of 2.96, indicating disapproval for the abuse domain. The violence domain was 11.46, with an SD of 4.41, indicating approval of the violence domain. The control domain was 10.05, with an SD of 3.10, indicating approval of the control domain. Finally, the total mean score for the IPVAS was 29.03 ± 8.14, indicating the negative attitude (disapproval) of nursing students toward IPVAS (see Table 2).

### Distribution of the intimate partner violence attitude scale (IPVAS) domains

**Fig. 1:**
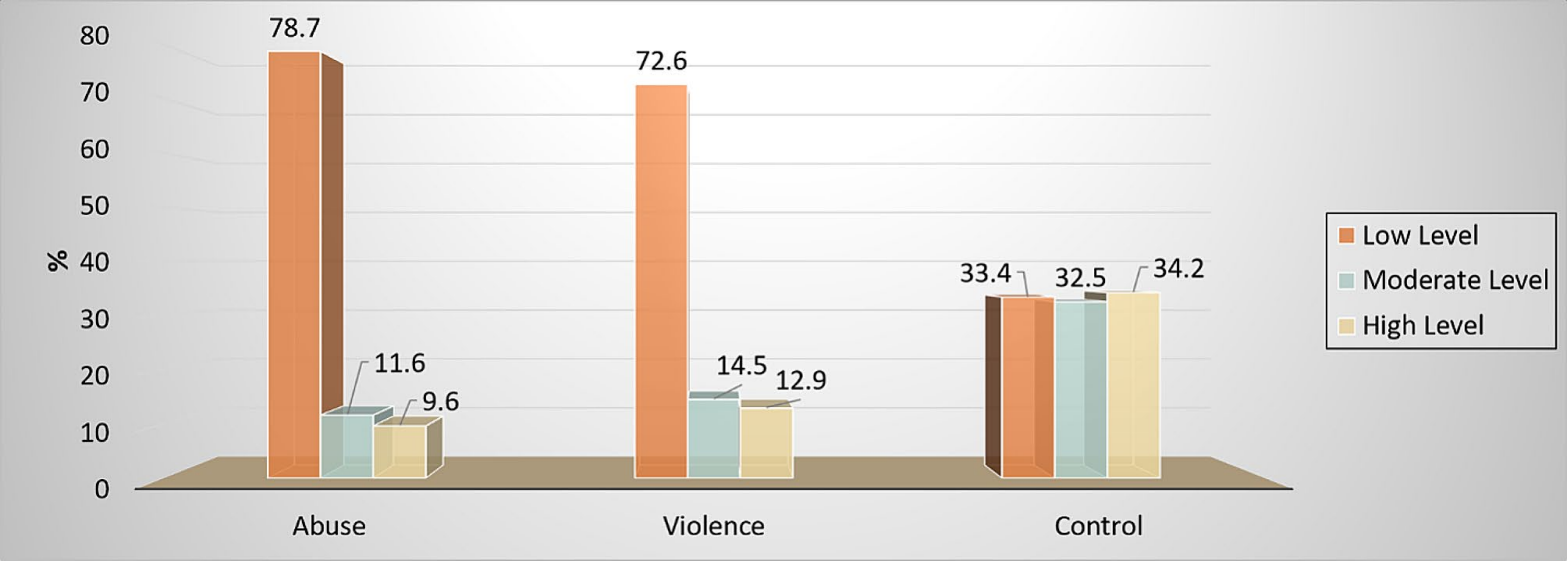
Distribution of the Intimate Partner Violence Attitude Scale (IPVAS) domains levels

Figure 1 shows the distribution of the Intimate Partner Violence Attitude Scale (IPVAS) domain levels among the students in this study. A total of 78.7% of nursing students had a low level of abuse, which means they disapproved of abuse. A total of 72.6% had a low level of violence domain of IPVAS, which means their approval of violence, and 34.2% had a high-level score of control domain, which means approval for control.

### Distribution of self-esteem levels for nursing students

The findings reveal that 12% of students had low self-esteem, 65.8% had a moderate level, and 22.2% had high self-esteem. The total score for self-esteem had a mean of 32.52, with a SD of 3.32. These findings suggest that nursing students had higher levels of self-esteem Table (3).

### Distribution of the general self-efficacy scale levels for nursing students

The results indicate that a significant portion of nursing students (62.8%) had a low level of self-efficacy. On the other hand, 29.7% had a moderate self-efficacy level; only 7.5% had a high self-efficacy level (Table 4).

### The correlation between the intimate partner violence attitude scale, self-esteem, and self-efficacy levels

The results showed a significant negative correlation between IPVAS and self-esteem (*r* = -0.065, *p* = 0.018*). This suggests that students with higher levels of self-esteem were less likely to approve of IPV. Additionally, there was a significant negative correlation between IPVAS and self-efficacy (*r* = -0.104, *p* = 0.005*). This finding suggests that students with higher levels of self-efficacy were less likely to approve of IPV (Table 5).

**Table 1:** Distribution of the sociodemographic characteristics of the nursing students

	N		%
**Age (Years)**			
18–20	476		36.0
21–23	846		64.0
**Mean ± SD**	20.65 ± 1.40		
**Gender**			
Female	738		55.8
Male	584		44.2
**Academic Year**			
1st	316		23.9
2nd	159		12.0
3rd	453		34.3
4th	394		29.8
**GPA**			
Below 2.00	5		0.4
2.00–2.32	86		6.5
2.33–2.99	92		7.0
3.00–3.66	733		55.4
3.67–4.00	406		30.7
**Marital status**			
Single	1237		93.6
Married	85		6.4
**Exposure to Violence**			
No	1018		77.0
Rarely	170		12.9
Yes	134		10.1
**Witnessing Violence**			
No	880		66.6
Rarely	232		17.5
Yes	210		15.9
**Type of Violence**			
None	871		65.9
Emotional	131		9.9
Physical	121		9.2
Economic	33		2.5
Verbal	166		12.6

SD: Standard deviation∗Grade point average (GPA) out of 5

**Table 2:** The mean score of the intimate partner violence attitude sub domains scale (IPVAS) for nursing students

	Mean ± SD
Abuse	7.51 ± 2.96
Violence	11.46 ± 4.41
Control	10.05 ± 3.10
Total Score	29.03 ± 8.14

SD: Standard Deviation

**Table 3:** Distribution of self-esteem level for nursing students

	N	%
LowSelf-esteem	159	12.0
ModerateSelf-esteem	870	65.8
HighSelf-esteem	293	22.2
Total Score (Mean ± SD)	32.52 ± 3.32	

**Table 4:** Distribution of the general self-efficacy scale levels for nursing students

	N	%
Low Self Efficacy	830	62.8
Moderate Self Efficacy	393	29.7
High Self Efficacy	99	7.5
Total Score (Mean ± SD)	23.69 ± 2.10	

SD: Standard Deviation

**Table 5:** Correlation between the intimate partner violence attitude scale, self– esteem and self–efficacy

	Intimate Partner Violence Attitude Scale		Self–Esteem Level		Self-Efficacy Level	
	R	P	r	P	R	p
Intimate Partner Violence Attitude Scale			− 0.065	0.018*	− 0.104	< 0.001**
Self– Esteem	− 0.065	0.018*			0.057	0.040*
Self– Efficacy	− 0.104	< 0.001**	0.057	0.040*		

* Statistically significant p value at ≤ 0.05

### Linear regression on factors affecting the intimate partner violence attitude scale

These findings highlight the significance of self-esteem and self-efficacy in shaping attitudes toward intimate partner violence. The negative standardized coefficients suggest that higher levels of self-esteem and self-efficacy are associated with lower approval for intimate partner violence. Furthermore, the significant p values (0.030* &<0.001**) indicate that these relationships are statistically significant Table (6).

## Discussion

This study was carried out on a sample of Egyptian nursing students to determine nursing students’ attitudes toward intimate partner violence and its relationship with their self-esteem and self-efficacy. The present study found that nursing students had disapproving negative attitudes toward IPV, as shown by their lower mean total intimate partner violence attitude scale (IPVAS). This could be related to the findings in Table 6, suggesting that students with higher self-esteem and self-efficacy tend to have less support for intimate violence. This aligns with a study by Alhalal [35], which found that nurses generally hold negative attitudes toward intimate partner violence. Similarly, another study was conducted on Saudi student attitudes and preparedness concerning managing intimate partner violence. This indicated that their knowledge and attitude were neutral, indicating neither approval nor disapproval of IPV [36].

**Table 6:** Linear regression on factors affecting the intimate partner violence attitude scale

Model	Unstandardized Coefficients		Standardized Coefficients		
	B	Std. Error	Beta	t	Sig.
(Constant)	43.011	3.242		13.265	< 0.001**
Self–Esteem Level	− 0.145	0.067	− 0.059	-2.167	0.030*
Self–Efficacy Level	− 0.390	0.106	− 0.101	-3.680	< 0.001**

* Statistically significant p value at ≤ 0.05

The current results also revealed a significant relationship between male gender and a higher mean of IPVA, indicating male acceptance of IPV and a possible decrease in gender equality attitudes. This perspective is supported by research conducted by Yang [37]. They found that individuals with more gender-equitable attitudes have higher awareness, positive attitudes, lower tolerance, and an expanded definition of severe violent actions.

In the current study, physical violence (attitudes toward both actual and potential physical abuse) was high, which means approving and accepting intimate partner violence. This could be attributed to many factors. These factors include globalization, rapid changes in society and culture, and watching violence and crimes continuously through social media and satellite TV. One of those crimes was the most famous crime of killing a university student in a very cruel and wild way by her previous intimate partner in front of the university students at Mansoura University, Egypt. Additionally, there is an increase in violent movies that represent the hero as a violent person, which makes youth and young adults identify with him. This agrees with Uzochukwu and Anierobi [38], who reported that watching violent TV shows and movies and visiting other social media sites has a highly harmful impact on young people because it makes them more aggressive.

Students’ experiences with or exposure to violence may be a further risk factor for embracing physical violence. This is corroborated by the significant relationship between seeing violence and the rise in IPVA means, which shows that students are more likely to tolerate and approve of violent behavior (as shown in Table 5). A study congruent with this explanation by Anikwe [39] stated that the victim had a possibility of physical and mental health problems related to IPV, which could increase their violent response and approval ofit.

Moreover, the current study’s mean of the IPVA abuse domain was low. This subscale measured how acceptable it was to experience or display verbal and nonverbal abuse behavior, which included disapproving of abuse by nursing students. This could be accounted for because abuse can have an impact long after the violence has subsided. The effects of abuse tend to be cumulative over time, with more severe abuse having a more significant adverse effect on the partner’s physical and mental health [40].

According to the current study, more than three-fifths of the students had moderate self-esteem, and almost one-quarter had high self-esteem. Their excellent GPA can be one of the causes of such growth. The results specifically demonstrated a substantial correlation between high self-esteem and GPA. This finding is consistent with research conducted by Ibrahim [41] on nursing students at the University of Mosul in Iraq. In that study, it was shown that both male and female student respondents had a positive perception of their self-esteem. In contrast, the current result disagrees with a study conducted at Kafrelsheikh University, Egypt, on nursing students. The results revealed that over half of the sample had moderate or low self-esteem [42].

Additionally, the findings showed that female students had higher self-esteem than male students. These findings contrast with research conducted on Egyptian nursing students, which found that both male and female nursing students had moderate levels of self-esteem [43]. One explanation for that result is the presence of other factors that affect self-esteem, such as perceived prejudice, exposure to professional stress, and professional identity [44].

The current study found that almost two-thirds of nursing students have low self-efficacy. This implies that many nursing students may lack confidence in their ability to excel in academic and clinical settings. The study also revealed a statistically significant relationship between exposure to or witnessing violence and low self-efficacy, which may explain these results. The results also revealed the lowest self-efficacy level for students exposed to physical and economic violence. This justification agrees with a study by Rode [45]on young adolescents in Poland, which found that victims of physical abuse had the highest level of anxiety and low self-competence. This disagrees with a study conducted byAbu Sharour [46] during COVID-19 and illustrates that the students had a moderate level of self-efficacy. Additionally, the study disagrees with Athira [47], who found that more than half of the nursing students had high self-efficacy.

Finally, the findings highlight the significance of self-esteem and self-efficacy in shaping attitudes toward intimate partner violence. A statistically significant negative relationship exists between self-esteem, self-efficacy, and IPVA. Higher self-esteem and self-efficacy are associated with lower approval for intimate partner violence. As low self-esteem is a significant symptom of depression, the result agrees with a study byIbala [48], which indicated that attitudinal acceptance of IPV is associated with worsened depressive symptoms. Moreover, the current study results agree with a study byKim and Gray [49] and Zlotnick [50], whi**c**h revealed that women with higher levels of self-efficacy were more likely to leave relationships with IPV and disapprove of them entirely. However, this result disagrees with a study byWalsh [51], which revealed that self-efficacy did not significantly predict experiencing IPV.

### Implications

Educating students about healthy relationships while enhancing their self-efficacy and self-esteem through training programs and seminars. Providing nursing students with tools to handle power dynamics and promote nonviolent attitudes can create a safer healthcare workplace for professionals and patients. Healthcare organizations must prioritize solving these issues and take preventive steps to reduce intimate partner violence among their staff. Further research is needed to explore the factors that contribute to attitudes and levels of self-esteem and self-efficacy among nursing students. It would also be useful to study how education and training affect these attitudes and develop interventions to foster healthy intimate partner violence (IPV) attitudes.

## Conclusion

The study found that most nursing students had low levels of abuse, indicating that they are less likely to participate in or approve intimate partner violence. However, it is troubling because many nurses reported a high degree of violence and control, suggesting that interventions to address power dynamics within partnerships may be needed. The study highlights the importance of self-efficacy and self-esteem in influencing attitudes toward intimate partner violence. It was found that nursing students with higher self-esteem and self-efficacy disapproved of intimate partner violence, emphasizing the need to encourage these qualities in nursing students.

### Relevance to nursing

It is crucial to address intimate partner violence in the nursing profession. Nurses may play a critical role in stopping the cycle of abuse by providing them with the information and skills to recognize and handle intimate partner violence. Training programs should include complete teaching on recognizing the indications of intimate partner violence, effective communication tactics, and appropriate intervention measures. Furthermore, nurses should be trained on accessible options for patients and themselves, such as local hotlines, support groups, and counseling services. Nursing programs may better assist their students and enable them to make a difference in the lives of their patients by prioritizing teaching on intimate partner violence prevention.

### Limitations

Our study has some limitations. The questionnaire was long to some extent, and students were busy. A large number of the students did not complete their questionnaires; therefore, those cases were omitted. Hence, it was difficult to take an equal sample of each academic year to compare differences in students’ IPVA, self-esteem and self-efficacy. It was difficult to fully understand the complex interplay between IPVA, self-esteem and self-efficacy, as the study is cross-sectional. Hence, longitudinal studies and controlled experiments are necessary to provide more conclusive insights into the relationship.

## Data Availability

The datasets used during the study are available from the corresponding author upon request.

## Abbreviations

IPV: Intimate partner violence
